# Acyclic retinoid induces differentiation and apoptosis of murine hepatic stem cells

**DOI:** 10.1186/s13287-015-0046-9

**Published:** 2015-03-26

**Authors:** Hong-Bin Guan, Yun-Zhong Nie, Yun-Wen Zheng, Kazuya Takiguchi, Hong-Wei Yu, Ran-Ran Zhang, Bin Li, Tomonori Tsuchida, Hideki Taniguchi

**Affiliations:** Department of Regenerative Medicine, Graduate School of Medicine, Yokohama City University, Yokohama, Kanagawa 236-0004 Japan; Department of Advanced Gastroenterological Surgical Science and Technology, Faculty of Medicine, University of Tsukuba, Tsukuba, 305-8575 Japan; Department of Histology and Embryology, Harbin Medical University, Harbin, 150081 China; Oregon Stem Cell Center, Oregon Health and Science University, Portland, OR 97239 USA; Advanced Medical Research Center, Yokohama City University, Yokohama, Kanagawa 236-0004 Japan

## Abstract

**Introduction:**

The therapeutic potential of acyclic retinoid (ACR), a synthetic retinoid, has been confirmed in experimental and clinical studies. Therapeutic targets include precancerous and cancer stem cells. As ACR is also involved in developmental processes, its effect on normal hepatic stem cells (HpSCs) should be investigated for understanding the underlying mechanisms. Here, we examined effects of the acyclic retinoid peretinoin on fresh isolated murine HpSCs.

**Methods:**

We isolated c-kit^−^CD29^+^CD49f^+/low^CD45^−^Ter119^−^ cells from murine fetal livers using flow cytometry. To evaluate the effect of ACR, we traced clonal expansion and analyzed cell differentiation as well as apoptosis during the induction process by immunofluorescent staining and marker gene expression.

**Results:**

ACR dose-dependently inhibited HpSCs expansion. Stem cell clonal expansion was markedly inhibited during the culture period. Moreover, ACR showed a significant promotion of HpSC differentiation and induction of cellular apoptosis. The expression of stem cell marker genes, *Afp*, *Cd44*, and *Dlk*, was downregulated, while that of mature hepatocyte genes, *Alb* and *Tat*, and apoptosis-related genes, *Annexin V* and *Caspase-3*, were upregulated. Flow cytometry showed that the proportion of Annexin V-positive cells increased after ACR incubation compared with the control. Data obtained by immunofluorescent staining for albumin and Caspase-3 corroborated the data on gene expression. Finally, we found that ACR directly regulates the expression of retinoic acid receptors and retinoid X receptors.

**Conclusions:**

These findings indicate that ACR inhibits the clonal expansion of normal HpSCs *in vitro* and promotes the differentiation of immature cells by regulating receptors of retinoic acid.

**Electronic supplementary material:**

The online version of this article (doi:10.1186/s13287-015-0046-9) contains supplementary material, which is available to authorized users.

## Introduction

Retinoic acid, a natural derivative of the metabolism of vitamin A, is an essential component of cell–cell signaling in embryogenesis, growth, and differentiation [1]. Retinoic acids can directly enter the nucleus and regulate target genes via nuclear receptors, including retinoic acid receptors (RARs) and retinoid X receptors (RXRs) [2]. Early studies of vitamin A deficiency [3-5] and compound RAR mutations [6,7] have indicated that retinoic acids are essential for the development of several organs, including the hindbrain, spinal cord, heart, eye, skeleton, forelimb buds, lung, pancreas, and genitourinary tract [1]. Recent studies suggest that retinoic acid can work in a paracrine manner to control the differentiation of pluripotent cells [8,9].

Peretinoin is a novel synthetic acyclic retinoid (ACR), with a structure similar to that of natural retinoic acid, that can bind to retinoid nuclear receptors such as RARs and RXRs [10]. Clinical studies have found that ACR can significantly reduce the incidence of post-therapeutic hepatocellular carcinoma (HCC) recurrence and improve the survival rate of HCC patients [11-14]. A phase II/III randomized, placebo-controlled trial demonstrated that 600 mg/day peretinoin can reduce the risk of HCC recurrence or death by approximately 40% compared with placebo [15]. Moreover, ACR also inhibits the progression of adult T-cell leukemia by inactivating nuclear factor-kB [16] as well as pancreatic cancer by inhibiting Ras activation [17] *in vitro* and *in vivo*.

Therapeutic effects of ACR have been confirmed in basic research [18,19] and clinical studies [11,12]. It has been reported that ACR exhibited the anti-HCC mechanism through increasing cell apoptosis [19,20] and inhibiting cell proliferation [21,22]. Further, Yasuda and colleagues [19] found that ACR could induce the production of albumin and reduce AFP-L3 levels in hepatoma-derived cell lines. These data may indicate that ACR can promote HCC differentiation to exhibit the anti-HCC effect. Our previous studies have demonstrated that ACR can inhibit the development of precancerous cells in HCC models [23], while the role of ACR in normal hepatic stem cells (HpSCs) remains unclear.

During liver development, fetal HpSCs can be developed from the foregut endodermal cells, and they differentiate into liver cell lineage, including hepatocytes and cholangiocytes [24,25]. Our previous study has identified the population of fetal HpSCs as c-Kit^−^CD29^+^CD49f^+/low^CD45^−^Ter-119^−^ [26]. Retinoic acid has played a significant role in organ development [1], whereas the role of ACR in liver regeneration it is still unclear [27-29]. Furthermore, as hepatic carcinogenic stem cells exhibit characteristics similar to normal HpSCs [30], a comprehensive understanding of the role of ACR in normal HpSCs would enable us to explore the mechanisms and functions of ACR in liver development and regeneration as well as in hepatic carcinogenesis. Although hepatic stem/progenitor cells in the developing liver have been extensively characterized [26], it is still under debate whether HpSCs exist in adult tissue, especially in recent studies [31,32]. Although these cells may not represent an important source of regeneration *in vivo*, their expandability and hepatocytic potential still make them an attractive potential source of liver cells for regenerative medicine [24,25].

In this study, the murine fetal HpSC subpopulation, c-kit^−^CD29^+^CD49^+/low^CD45^−^Ter119^−^ cells, were isolated by flow cytometry as previously described [33]. And we examined the effects of ACR on fetal HpSCs, including clonal colony expansion, differentiation, and apoptosis as well as on retinoic acid-related receptors.

## Materials and methods

### Animals and ethics

Embryonic day 13.5 C57BL/6 J mice were purchased from Japan SLC Inc. (Shizuoka, Japan). Animal experimental work was conducted in accordance with the Guidelines for Proper Conduct of Animal Experiments (Science Council of Japan), and all experimental procedures were approved by the institutional review board of Animal Research Center, Yokohama City University School of Medicine (No.11–68).

### Hepatic stem cell isolation

Suspended liver cells were obtained from embryonic day 13.5 C57BL/6 fetal mice as previously described [33]. In brief, dissociated liver cells were stained with biotinylated anti-CD45 and Ter-119 monoclonal antibodies, anti-CD29-FITC antibody, anti-CD49f-PE antibody, and anti-c-Kit-APC antibody. Cells positive for the biotinylated antibodies were detected with streptavidin-labeled allophycocyanin-Cy7 (all monoclonal antibodies were purchased from BD PharMingen, San Diego, CA, USA). Labeled cells were then analyzed and separated using MoFlo (DakoCytomation, Denmark) and Summit version 4.0 software (DakoCytomation, Denmark).

### Cell culture

Sorted cells were seeded on type IV collagen-coated dishes (Becton Dickinson, San Jose, CA, USA) using our standard culture medium. Williams’ Medium E (Gibco, Grand Island, NY, USA) was supplied with 10% fetal bovine serum (MP Biomedicals, LLC, Solon, OH, USA), 1 μg/mL insulin (Wako, Osaka, Japan), 1 × 10^−7^ M dexamethone (Sigma, St Louis, MO, USA), 10 mmol/L nicotinamide (Sigma), 2 mmol/L L-glutamine (Gibco), 50 mmol/L HEPES (Wako), 50 mmol/L β-mercaptoethanol (Sigma), 50 ng/mL hepatocyte growth factor (Sigma), 20 ng/mL epidermal growth factor (Sigma), and 100 U penicillin/streptomycin (Gibco). Cells were cultured at 37°C in a humidified atmosphere of 5% CO_2_.

ACR as peretinoin was provided by Kowa (Tokyo, Japan), and resolved in dimethyl sulfoxide (DMSO). For the half maximal inhibitory concentration (IC50) experiment, the medium was supplemented with ACR after 24 hours of culture, and the final concentrations were 0, 0.5, 1.0, 2.0, 4.0, and 8.0 mg/L. The same concentration of DMSO was used in the controls. The medium was changed every other day. After 7 days of culture, cell number and colonies were counted. After determination of the IC50 dose, all following experiments were supplemented with this dose.

For assessing the colony expansion and proliferation of HpSCs, the IC50 dose (3.5 mg/L ACR with 0.05% DMSO) was added to cultures after 24 hours. The same concentration of DMSO was used in the controls. Single cell growth was traced at days 0, 1, 3, 5, and 7. The colony size and the frequency of colony formation were traced and calculated after 7 days of culture (colonies containing >10 cells were counted).

For experiments on the vacuolar degeneration of HpSCs, 3.5 mg/L ACR with 0.05% DMSO was added to cultures after 72 hours, and the formation of vacuolar cells was detected after 24 hours of incubation with ACR. The same concentration of DMSO was used in the controls.

For the other experiments, 24 cultures were supplemented with the IC50 dose (3.5 mg/L ACR with 0.05% DMSO) with the same concentration of DMSO used in the controls. After 5 days of culture, cells were harvested for immunofluorescence studies and RNA isolation.

### Immunofluorescence

Culture cells were fixed with 4% paraformaldehyde in a 6-well plate at 4°C for 15 minutes, washed three times with phosphate-buffered saline containing 0.05% Tween 20 (Wako), blocked with 10% normal goat serum, followed by overnight incubation with Alb (Biosciences, Cambridge, UK), Ki67 (Abcam, Cambridge, MA, USA), or Cleaved Caspase-3 (Asp175) Antibody (Cell Signaling Technology, Beverly, MA, USA). Next, cells were incubated with 488- or 555-labeled secondary antibodies for 2 hours. After the final washing, nuclei were stained blue with DAPI and viewed with a Zeiss Axio Imager.M1 microscope (Carl Zeiss, Germany).

### RNA isolation and real-time polymerase chain reaction

Total RNA was isolated using ISOGEN (Nippon gene, Tokyo, Japan) according to the manufacturer’s instructions. A total of 1 μg RNA was used as the template for single strand cDNA synthesis utilizing random primers and the SuperScriptIII Reverse Transcriptase (Invitrogen, Carlsbad, CA, USA). cDNA was amplified using SYBR green PCR Mix (Takara, Otsu, Japan) on the ABI PRISM 7900 real-time PCR system (Applied Biosystems, Foster City, CA, USA), programmed for 95°C for 2 minutes, followed by 40 cycles of 95°C for 10 seconds, and 60°C for 50 seconds. Amplification results were analyzed using RQ Manager 1.2 (Applied Biosystems) and the gene of interest was normalized to the corresponding 18S results. An additional primer sequence lists this in more detail (see Additional file 1).

### Apoptosis analysis

After 5 days of culture, mouse fetal HpSCs were harvested with 0.05% trypsin (Sigma). Cells were washed with phosphate-buffered saline and cellular apoptosis was detected by Annexin-V FLUOS staining kit (Roche Diagnostics, Indianapolis, IN, USA) according to the manufacturer’s instructions. Samples were stained with 10 μg/mL propidium iodide in the dark at 4°C for 10 minutes. Annexin V-positive cells were analyzed with MoFlo and the Summit version 4.0 software (both DakoCytomation).

### Statistical evaluation

Values are expressed as mean ± SD. Statistical analysis was performed using Mann–Whitney *U* test to compare the mean values between two groups. Values of *P* < 0.05 are considered to be statistically significant.

## Results

### Hepatic stem cell isolation and dose-dependent response to acyclic retinoid

**Figure 1:**
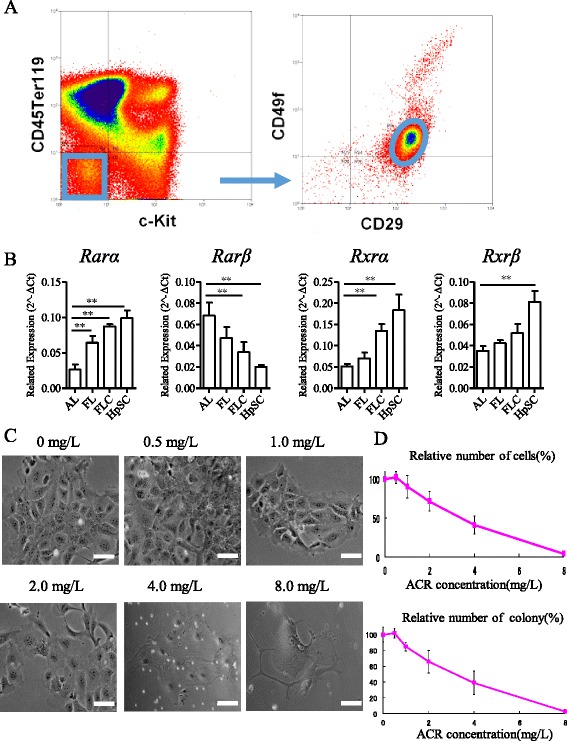
**Hepatic stem cell isolation and dose-dependent response to acyclic retinoid. (A)** Hepatic stem cells (HpSCs) were sorted by flow cytometry using the gate c-Kit^−^CD29^+^CD49f^+/low^CD45^−^Ter-119^−^. **(B)** The expression of *Rarα*, *Rarβ*, *Rxrα*, and *Rxrβ* in adult liver (AL; n = 3), fetal liver (FL; n = 3), fetal liver cell (FLC; n = 3), and HpSCs (n = 3). **(C)** Acyclic retinoid (ACR) inhibited the growth of HpSCs with different concentrations (0, 0.5, 1.0, 2.0, 4.0, and 8.0 mg/L). Scale bar = 200 μm. **(D)** Relative numbers of HpSCs and numbers of HpSC colonies in different concentrations of ACR after 7 days of culture (n = 3). Data are shown as means ± SD. Mann–Whitney *U*-test. *P < 0.05; **P < 0.01.

To investigate the role of ACR on HpSCs, we sorted HpSCs from embryonic day 13.5 fetal mouse liver with HpSC markers as reported (c-Kit^−^CD29^+^CD49f^+/low^CD45^−^Ter-119^−^) (Figure 1A), as such cells show strong self-renewal and colony formation capabilities (Figure 1C). ACR exerts its function through the retinoid-related receptors, including RARs and RXRs. As shown in Figure 1B, the expression of RARs (*Rarα* and *Rarβ*) and RXRs (*Rxrα* and *Rxrβ*) could be detected in HpSC, except *Rarγ* and *Rxrγ*. Compared with adult liver, HpSCs showed a higher expression of *Rarα*, *Rxrα*, and *Rxrβ* but a lower expression of *Rarβ*. To determine the role of ACR on HpSCs, we treated HpSCs with different concentrations of ACR, including 0, 0.5, 1.0, 2.0, 4.0, and 8.0 mg/L. After 7 days of culture, the numbers of total cells and colonies had significantly decreased in the cell population incubated with ACR, and the decrease was dose dependent (Figure 1C and D). The IC50 of ACR on HpSCs was calculated to 3.5 mg/L.

### Acyclic retinoid inhibits clonal expansion and proliferation of hepatic stem cells

**Figure 2:**
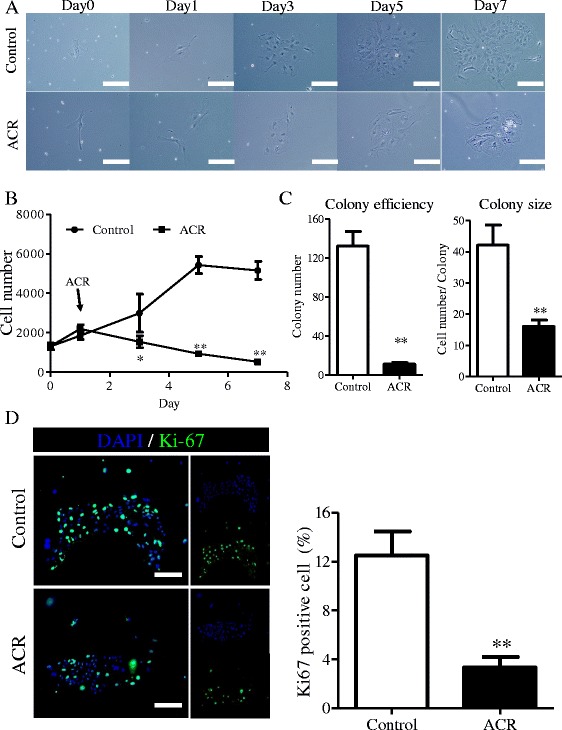
**Acyclic retinoid inhibits clonal expansion and proliferation of hepatic stem cells. (A)** Tracing colony expansion of hepatic stem cells (HpSCs) with half maximal inhibitory concentration dose (3.5 mg/L) of acyclic retinoid (ACR) during 7 days of culture. **(B)** Tracing the proliferation of HpSCs during 7 days of culture treated with ACR (n = 6). **(C)** The number and size of colonies formed at day 5 of ACR treatment (n = 6). **(D)** Immunofluorescence for Ki-67 after treatment with ACR, and statistical data of percentage of Ki-67 positive cells (n = 6). Scale bar = 200 μm. Data are shown as means ± SD. Mann–Whitney *U*-test. *P < 0.05; **P < 0.01.

To further evaluate the role of ACR on HpSCs, we used the IC50 dose of ACR to trace the clonal expansion of HpSCs. As shown in Figure 2A and B, HpSCs demonstrated high self-renewal capacity and colony formation capability; the colony-forming efficiency was as high as 1.32% (with values up to 132.16 ± 15.02 per 10,000 plated cells), with large average colony sizes, including 42.18 ± 6.50 cells per colony (Figure 2C), while the self-renewal and colony formation capacities were significantly inhibited by ACR. After ACR treatment, the clonal expansion of HpSCs almost stopped, the colony number decreased to 8.4% compared to the control (11.11 ± 1.59 per 10,000 plated cells), and colony sizes were only 38% the size of the control, with 16.15 ± 1.98 cells per colony (Figure 2C). To probe the role of ACR in the inhibition of clonal expansion, we examined the Ki-67-positive cells after incubation with ACR. After incubation with ACR, the Ki-67-positive cells in HpSC-derived colonies significantly decreased from 12.51 ± 1.95% to 3.35 ± 0.86% (Figure 2D). To further confirm the inhibition of colony expansion and proliferation by ACR, we analyzed the expression of the cell cycle-related genes, *p21*^*cip1*^ and *Cyclin D1*. The expression of *p21*^*cip1*^ increased by 6.8-fold in ACR-treated cells (see Additional file 2), and hence were clearly different in gene expression (*P* < 0.05), while the expression of *Cyclin D1* was significantly downregulated (see Additional file 2).

### Acyclic retinoid promotes differentiation of hepatic stem cells

**Figure 3:**
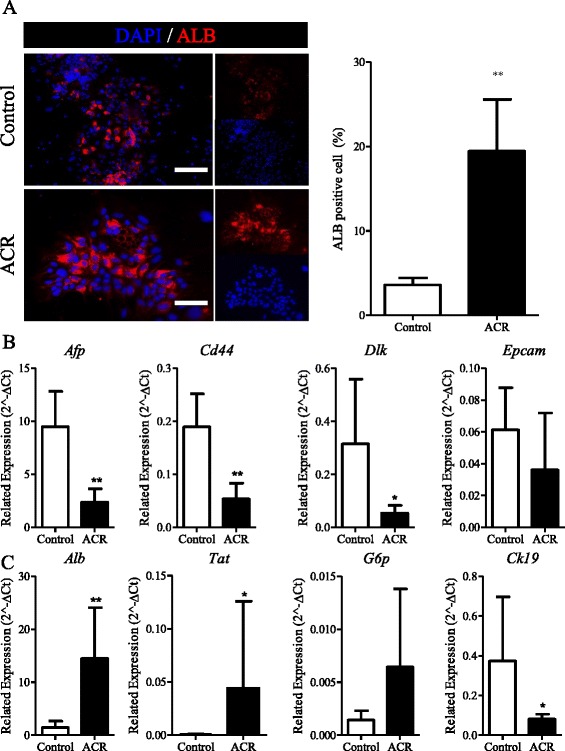
**Acyclic retinoid promotes differentiation of hepatic stem cells. (A)** Immunofluorescence for albumin (ALB) after treatment with acyclic retinoid (ACR), and percentage of ALB-positive cells (n = 6). Scale bar = 200 μm. **(B)** Real-time PCR analysis of the relative mRNA expression of *Afp*, *Cd44*, *Dlk*, and *Epcam* after treatment with ACR (n = 5 to 8). **(C)** Real-time PCR analysis of the relative mRNA expression of *Alb*, *G6p*, *Ck19*, and *Tat* after treatment with ACR (n = 3 to 8). Data are shown as means ± SD. Mann–Whitney *U*-test. *P < 0.05; **P < 0.01.

To investigate the role of ACR in colony expansion and proliferation, we hypothesized that ACR can induce the differentiation of HpSCs. After 5 days of culture, 3.60 ± 0.82% HpSCs were albumin positive (Figure 3A), while the percentage among ACR-treated cells was 19.44 ± 6.13% (Figure 3A). The related gene expression also indicated that ACR promotes the differentiation of HpSCs. The HpSC marker genes, including *Afp*, *Cd44*, and *Dlk*, were significantly downregulated by ACR (Figure 3B), and the mature hepatic genes, *Alb* and *Tat,* were significantly upregulated after ACR treatment (Figure 3C). HpSCs can also differentiate into bile duct cells, while the expression of the bile duct cell marker gene, *Ck-19*, was inhibited by ACR (Figure 3C). These data indicate that ACR promotes the hepatocellular but not cholangiolar differentiation of HpSCs.

### Acyclic retinoid induces cellular apoptosis of hepatic stem cells

**Figure 4:**
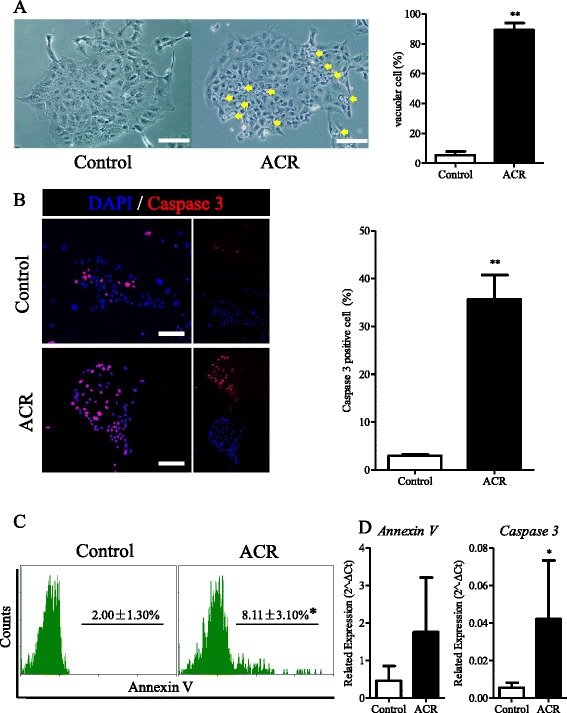
**Acyclic retinoid induces cellular apoptosis of hepatic stem cells. (A)** Observation of the formation of vacuolar cells after 24 hours of incubation with acyclic retinoid (ACR), and the ratio of vacuolar cells (n = 9). **(B)** Immunofluorescence for Caspase 3 after incubation with ACR, and the ratio of Caspase 3-positive cells (n = 6). **(C)** Flow cytometry analysis of Annexin V-positive cells after incubation with ACR (n = 3). **(D)** Real-time PCR analysis of the relative mRNA expression of *Annexin V* and *Caspase 3* after incubation with ACR (n = 5–8). Scale bar = 200 μm. Data are shown as means ± SD. Mann–Whitney *U*-test. *P < 0.05; **P < 0.01.

Apoptosis can be another cause of the inhibition of clonal expansion. Twenty-four hours after adding ACR, we observed the appearance of vacuolar cells (Figure 4A; approximately 89.17 ± 4.79% of the cells were vacuolar), while only 5.42 ± 2.52% of the cells not incubated with ACR were vacuolar (Figure 4A). ACR significantly induced the vacuolar cell formation in HpSCs. Caspase 3 is known as a crucial mediator of apoptosis [34]. As shown in Figure 4B, we found that ACR markedly elevated the percentage of Caspase 3-positive cells from 2.96 ± 0.27% to 35.68 ± 5.05%. To further confirm the apoptosis process induced by ACR, we analyzed the relative distribution of Annexin V-positive cells with or without ACR incubation. Without ACR incubation, Annexin V-positive cells only amounted to 2.00 ± 1.30%, while the percentage significantly increased to 8.11 ± 3.10% after ACR incubation (Figure 4C). Moreover, we compared the expression of apoptosis-related genes, *Caspase 3* and *Annexin V*, with and without ACR incubation. After incubation with ACR, *Caspase 3* was shown to be 7.55-fold enhanced, indicating a significant difference in the expression (Figure 4D). The increased expression of *Annexin V* in ACR-treated cells was not significant (Figure 4D).

### Acyclic retinoid regulates *Rars* and *Rxrs* expression in hepatic stem cells

**Figure 5:**
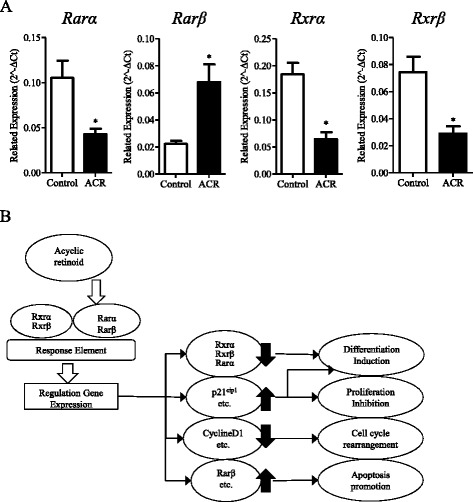
**Acyclic retinoid regulates** ***Rars*** **and** ***Rxrs*** **expression in hepatic stem cells. (A)** Real-time PCR demonstrated the relative mRNA expression of *Rarα*, *Rarβ*, *Rxrα*, and *Rxrβ* in hepatic stem cells (HpSCs) after incubation with or without acyclic retinoid (ACR; n = 3). **(B)** Schematic model for the role of ACR in HpSCs. ACR binds to the retinoic acid receptor (RAR) thereby transactivating genes through the retinoic acid responsive element, or by binding to the retinoid X receptor (RXR) and transactivating genes via the retinoid X responsive element. Subsequently, ACR induces differentiation, inhibits proliferation, arrests cell cycle, and promotes apoptosis in HpSCs through the related gene regulation. Furthermore, ACR regulates the receptor genes, *Rarα*, *Rarβ*, *Rxrα*, and *Rxrβ* and enhances ACR action. Data are shown as means ± SD. Mann–Whitney *U*-test. *P < 0.05; **P < 0.01.

ACR exerts its functions through the retinoid-related receptors, RARs and RXRs; therefore, the regulation of RARs and RXRs could also influence the function of ACR. We previously found that HpSCs exhibit the expression of *Rarα*, *Rarβ*, *Rxrα,* and *Rxrβ* (Figure 1B). In order to test the hypothesis that these receptors are regulated by ACR, performing special functions on HpSCs, we treated HpSC-derived colonies with ACR, and quantitated the expression of *Rarα*, *Rarβ*, *Rxrα*, and *Rxrβ*. As shown in Figure 5A, we found that ACR clearly inhibited the expression of *Rarα*, *Rxrα*, and *Rxrβ*, by a 2.45-, 2.86-, and 2.56-fold decrease, respectively. However, the expression of *Rarβ* was significantly enhanced by ACR, showing a 3.04-fold increase. The considered schematic model for the role of ACR in HpSCs is summarized and profiled in Figure 5B.

## Discussion

A previous study reported that ACR accelerated liver regeneration following partial hepatectomy in a rat model [29], and it was observed that hepatocyte DNA synthesis was enhanced by ACR. All-trans retinoic acid (ATRA), a natural retinoid, was also shown to accelerate liver regeneration through the induction of the cell cycle genes (*Cyclin A*, *Cyclin B*, *Cyclin D*, and *Cyclin E*), and increase the proliferation of hepatocytes [35]. In the present study, we focused on the role of the ACR peretinoin on fresh isolated HpSCs in developing livers. We found that ACR inhibits clonal expansion and induces the differentiation of HpSC-derived colonies accompanied by the downregulation of the cell cycle-related gene, *Cyclin D1*. This difference in the role of ACR and ATRA in cell cycle regulation and DNA synthesis results from the biphasic dose–response relationship of ACR. In fact, low-dose ACR promotes hepatocyte proliferation and DNA synthesis and stimulates liver regeneration, whereas higher doses actually reduce liver regeneration and decrease DNA synthesis. In contrast, ATRA dose-dependently stimulates liver regeneration [29]. Although ACR shares some characteristics with natural retinoids *in vitro* and *in vivo*, it has been reported that ACR differs in several biological effects from ATRA [36-38]. ACR might mediate different mechanisms of action to that of the natural retinoids in stimulating normal hepatocyte proliferation. However, this still remains to be elucidated.

ACR is known as a powerful agent in the clinical therapy of HCC through increasing cell apoptosis [19,20], induction of cell differentiation [19,39] and suppression of cellular proliferation [21,22]. Our data show that ACR could also induce cellular apoptosis, promote the differentiation into hepatocytes, and inhibit clonal expansion and proliferation. The schematic model is presented as Figure 5B. The precise mechanism of ACR-induced apoptosis is still not well understood. In part, the theory is that nuclear retinoid receptors are ligand-dependent transcription factors that bind to RAR or RXR elements in the promoter regions of retinoid responsive target genes [2]. There is an increase in the RARβ and p21CIP1 proteins and their corresponding mRNAs and a decrease in the hyperphosphorylated form of the retinoblastoma protein and subsequently a decrease in both cyclin D1 protein and mRNA. After inhibition of the transcriptional activity of the cyclin D1, c-fos, and activator protein promoters, there is an increase of cells in G0-G1 phase and apoptosis is induced [40]. RARβ might be an intermediate in a signaling cascade induced by retinoic acid which ultimately activates a tumor-specific apoptosis program mediated by tumor necrosis factor-related apoptosis-inducing ligand [41]. However, given the fact that RARβ2 is suppressed at early stages of carcinogenesis, agonists will be useful only in combination with agents able to reverse the silencing of RARβ2 [41]. In addition, it is also reported that polyinosinic:polycytidylic acid-mediated activation of Toll-like receptor 3 (TLR3) induces microRNAs targeting DNA methyltransferases, leading to demethylation and re-expression of RARβ [42]. As a result, cancer cells become sensitive to retinoic acid and undergo apoptosis both *in vitro* and *in vivo* [42]. This study provides evidence of an antitumoral mechanism of action upon TLR3 activation and the biological rationale for a combined TLR3 agonist/retinoic acid treatment of prostate and breast cancer [42].

Furthermore, ACR could perform similar functions on HpSCs in HCC. The similarity may suggest a close relationship between HpSCs, HCC, and liver cancer stem cells. Evidence exists that normal stem cells can be reprogrammed into cancer stem cells [43]. Chiba and colleagues reported that HCC could be initiated from normal HpSCs by the overexpression of the *Bmi-1* gene [44]. Moreover, our previous study has identified precancerous cells in rat hepatic oval cells fraction, and ACR directly prevents *de novo* HCC by inhibiting the development of the precancerous stem cells [23]. Since normal stem cells shared some common characteristics with precancerous stem cells with regard to their response to ACR, the result may indicate that ACR not only exhibits the anti-tumor factor in HCC but also exerts the inhibition on tumor original cells, liver cancer stem cells, and precancerous cells. Researchers hypothesized that ACR inhibits the development of HCC through the “clonal deletion” method [10]. Our data show that the clonal expansion of HpSCs was inhibited by ACR and thereby partly verifies the “clonal deletion” hypothesis.

Retinoids exert their biological effects through distinct retinoid-related receptors. ATRA and 13-cis-retinoic acid are likely to act mainly through RARβ [45]; 9-cis-retinoic acid always activates RXRα [46] and ACR has been reported to exert its biological function through RARβ [22,47]. Moreover, ACR increased the expression of *RARβ* in normal cells and HCCs, and induced apoptosis, while ACR did not regulate the expression of other retinoid-related receptors [22,47]. In the present study, we also observed the different expression of *Rars* and *Rxrs* in adult liver and HpSCs. These results indicate that the expression of *Rars* and *Rxrs* are related to the degree of differentiation. Compared with adult hepatic cells (highly differentiated), HpSCs (poorly differentiated) show a higher expression of *Rarα*, *Rxrα*, and *Rxrβ* and a lower expression of *Rarβ*. Abnormalities of RARs and RXRs are also related to HCC [48,49]. The expression of *RARα* and *RXRα* were clearly increased [48,49] and *RARβ* was markedly suppressed in human HCC and HCC cell lines [48]. Moreover, we found that *RARα*, *RXRα*, and *RXRβ* showed higher expression, while *RARβ* showed lower expression in colon carcinoma cells (poorly differentiated), the normal colon (highly differentiated) being the control (data not shown). ACR could regulate the expression of *Rars* and *Rxrs* in HpSCs. After incubation with ACR, the expression of *Rarβ* was promoted and apoptosis was induced in HpSCs and the derived colonies. ACR can inhibit the expression of *Rarα*, *Rxrα*, and *Rxrβ*; it exhibits a special effect on HpSCs to promote the differentiation of HpSC-derived cells through regulated *Rars* and *Rxrs*. Immature cell types might respond to ACR if its expression is high for the receptors no matter whether they are from the normal or diseased tissue; this should contribute to the understanding of the mechanism of action of ACR and enhance or relate to treatments in other immature tissues, such as other cancerous or precancerous tissues. This may suggest that ACR is an effective drug candidate in clinical colon carcinoma therapy. Moreover, the expression of *Rars* and *Rxrs* would be diagnostic markers applicable to ACR in clinical carcinoma therapy.

## Conclusions

In this study, we found that ACR can regulate the expression of related genes to inhibit clonal expansion and proliferation, promote differentiation, and induce apoptosis in HpSCs. Moreover, ACR can regulate the expression of *Rars* and *Rars* in HpSCs, and *Rars* and *Rxrs* may directly affect proliferation, differentiation, and apoptosis in HpSCs (Figure 5B). In conclusion, ACR promoted the differentiation of HpSCs, and ACR could also induce apoptosis in HpSCs and in smooth muscle cells [47] through *Rars* and *Rxrs*. Future studies on the roles of *Rars* and *Rxrs* will help to further explore the application of ACR in clinical carcinoma therapy.

## Additional files

Additional file 1:
**Primers used in quantitative RT-PCR.**
Additional file 2:
**RT-PCR analysis of the relative mRNA expression of cyclin D1 and p21** ^**cip1**^ **after treatment with acyclic retinoid (ACR) (n = 3 to 5).**

## Abbreviations

ACR: acyclic retinoid
ATRA: all-trans retinoic acid
DMSO: dimethyl sulfoxide
HCC: hepatocellular carcinoma
HpSC: hepatic stem cell
IC50: half maximal inhibitory concentration
RAR: retinoic acid receptor
RXR: retinoid X receptor
TLR3: Toll-like receptor 3
