# Supplementary material for: Acyclic retinoid induces differentiation and apoptosis of murine hepatic stem cells
Source: Stem Cell Res Ther. 2015 Mar 26;6(1):51. doi: 10.1186/s13287-015-0046-9 (PMC4417297; doi:10.1186/s13287-015-0046-9)
Supplement: Additional file 1: — Primers used in quantitative RT-PCR. [file 13287_2015_46_MOESM1_ESM.pdf]

Additional file 1. Primers used in quantitative RT-PCR

Gene	Forward (5‘—3‘)	Reverse (5‘—3‘)
Afp	CTCAGCGAGGAGAAATGGTC	GAGTTCACAGGGCTTGCTTC
Alb	GATTCTGACCCAGTGTTGTG	CTGGAGCACTTCATTCTCTG
Annexin V	GACTTCCCTGGATTTGATGG	TTCGGGATGTCAACAGGTTC
Caspase 3	GGCGACTACTGCCGGAGTCTGA	GCCACAGTCCAGCTCCGTACC
Cd44	AACCAGGACAGTGGAGTGAC	TCCTACTATTGACCGCGATG
Ck19	GACCCTCCCGAGATTACAAC	TTCTGAAGTCATCTGCAGCC
Cyclin D1	AGAAGGAGATTGTGCCATCC	CACTTCTGCTCCTCACAGACC
Dlk1	CAATGGAGTCTGCAAGGAAC	AGCATTCGTACTGGCCTTTC
Epcam	AGAATACTCTGTCATTTGCTCCAAACT	GTTCTGGATGCCCCTTC
G6p	GTGGGCATCAATCTCCTCTG	TGTCCAGGACCCACCAATAC
ki67	CCAGCTGTCCTCAAGACAATC	GCGTCTTTGATCATTTGTCCTC
$p21^{cip1}$	GATCCACAGCGATATCCAGAC	GGACATCACCAGGATTGGAC
Rarα	TGGCATCCTCTTTGATACCC	GTCCCAAGAATCCGTCCTTT
Rarβ	ATGAATAACCAGGCCTCACG	GCAAGGAGAAGCTTCCACAC
Rxrα	GATATCAAGCCGCCACTAGG	CAGATGTGCTTGGTGAAGGA
Rxrβ	TCTGAATTTGTCGGGGTCTC	GCCAAATGAGAAGGAAGCAG
Tat	TCTACAGGACATTGGCTGAG	GTTGTTGACCACGAGACAAG
