# Supplementary material for: Acyclic retinoid induces differentiation and apoptosis of murine hepatic stem cells
Source: Stem Cell Res Ther. 2015 Mar 26;6(1):51. doi: 10.1186/s13287-015-0046-9 (PMC4417297; doi:10.1186/s13287-015-0046-9)
Supplement: Additional file 2: — RT-PCR analysis of the relative mRNA expression of cyclin D1 and p21 cip1 after treatment with acyclic retinoid (ACR) (n = 3 to 5). [file 13287_2015_46_MOESM2_ESM.pdf]

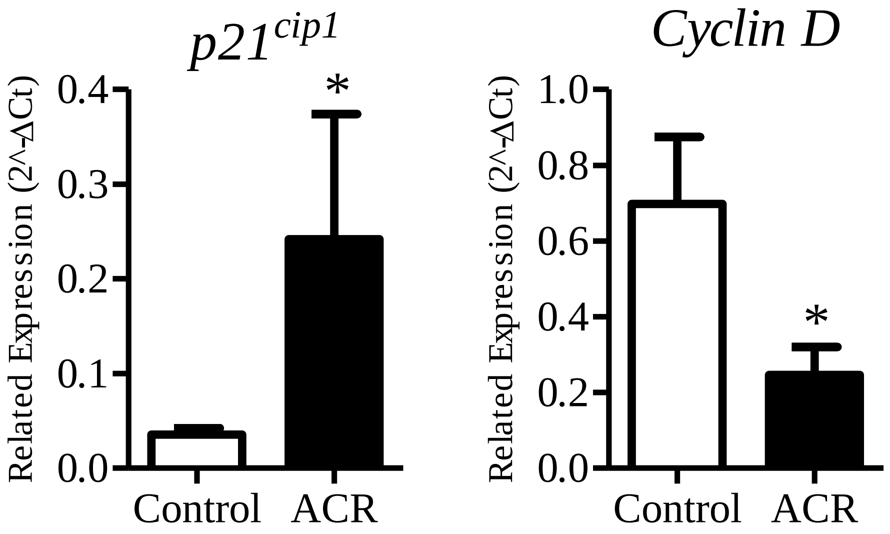

Additional file 2. Real-time PCR analysis of the relative mRNA expression of Cyclin D1 and p21cip1 after treatment with ACR (n = 3–5).
